# Interactions of the N- and C-Terminal SH3 Domains of *Drosophila* Drk with the Proline-Rich Peptides from Sos and Dos

**DOI:** 10.3390/ijms241814135

**Published:** 2023-09-15

**Authors:** Pooppadi Maxin Sayeesh, Mayumi Iguchi, Yusuke Suemoto, Jin Inoue, Kohsuke Inomata, Teppei Ikeya, Yutaka Ito

**Affiliations:** Department of Chemistry, Tokyo Metropolitan University, 1-1 Minami-Osawa, Hachioji, Tokyo 192-0373, Japan; sayeesh@tmu.ac.jp (P.M.S.); iguchi-mayumi@ed.tmu.ac.jp (M.I.); jinoue@megabank.tohoku.ac.jp (J.I.); kinomata@tmu.ac.jp (K.I.)

**Keywords:** Drk, Sos, Dos, SH3 domain, proline-rich motif

## Abstract

Drk, a homologue of human GRB2 in *Drosophila*, receives signals from outside the cells through the interaction of its SH2 domain with the phospho-tyrosine residues in the intracellular regions of receptor tyrosine kinases (RTKs) such as Sevenless, and transduces the signals downstream through the association of its N- and C-terminal SH3 domains (Drk-NSH3 and Drk-CSH3, respectively) with proline-rich motifs (PRMs) in Son of Sevenless (Sos) or Daughter of Sevenless (Dos). Isolated Drk-NSH3 exhibits a conformational equilibrium between the folded and unfolded states, while Drk-CSH3 adopts only a folded confirmation. Drk interacts with PRMs of the PxxPxR motif in Sos and the PxxxRxxKP motif in Dos. Our previous study has shown that Drk-CSH3 can bind to Sos, but the interaction between Drk-NSH3 and Dos has not been investigated. To assess the affinities of both SH3 domains towards Sos and Dos, we conducted NMR titration experiments using peptides derived from Sos and Dos. Sos-S1 binds to Drk-NSH3 with the highest affinity, strongly suggesting that the Drk-Sos multivalent interaction is initiated by the binding of Sos-S1 and NSH3. Our results also revealed that the two Sos-derived PRMs clearly favour NSH3 for binding, whereas the two Dos-derived PRMs show almost similar affinity for NSH3 and CSH3. We have also performed docking simulations based on the chemical shift perturbations caused by the addition of Sos- and Dos-derived peptides. Finally, we discussed the various modes in the interactions of Drk with Sos/Dos.

## 1. Introduction

Adapter proteins and their Src homology 3 (SH3) domains play crucial roles in signal transduction pathways that regulate various cellular processes, including cell proliferation, differentiation, and apoptosis [1,2]. The SH3 domain is a small protein module consisting of approximately 60 amino acids, adopting a characteristic 3D structure comprising five β-strands [3,4]. The binding of SH3 domains with proline-rich motifs (PRMs) is crucial for the efficient transmission of signals in the signal transduction pathways. These interactions have garnered significant attention in clinical research for developing inhibitors that can interrupt the interactions, ultimately preventing the development of tumours [5]. 

Among adaptor proteins, human growth factor receptor-bound protein 2 (GRB2) has been widely investigated to understand its functions [6]. In the RAS signalling pathway, GRB2 transmits growth factor signals downstream in response to, e.g., epidermal growth factor receptor (EGFR) activation [7], and further establishes a link between an active receptor tyrosine kinase (RTK) and RAS-specific guanine nucleotide exchange factor (GEF), such as Son of Sevenless homologue 1 (SOS1) at the plasma membrane [8]. Besides SOS1, GRB2 is known to interact with a variety of proteins, including GAB1 [9] and GAB2 [10] (GRB2-associated binders 1 and 2).

**Figure 1:**
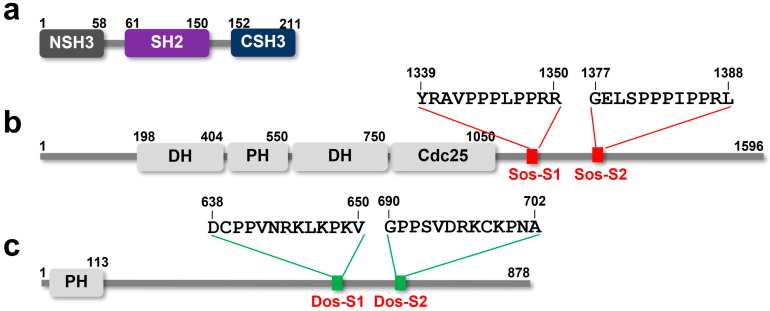
Domain structures of *Drosophila* Drk (**a**), Sos (**b**) and Dos (**c**). In (**b**,**c**), the positions of proline-rich motifs Sos-S1, Sos-S2, Dos-S1 and Dos-S2 are indicated.

Downstream receptor kinase (Drk) is a *Drosophila* homologue of GRB2, containing 211 amino acid residues [11]. As with GRB2, Drk consists of three domains, one Src homology domain 2 (SH2) flanked by two SH3 domains (NSH3 and CSH3, respectively) (Figure 1a). Drk and the RTK Sevenless (Sev) functionality necessitates the specification of photoreceptor R7 in the *Drosophila* eye [11]. When Sev is activated, Drk binds specifically towards the phospho-tyrosine residue (Y2546) via its SH2 domain, while NSH3 and CSH3 bind with the C-terminal intrinsically disordered region (IDR) of Son of Sevenless (Sos). Additionally, it has also been reported that Drk binds the C-terminal IDR of Daughter of Sevenless (Dos), a homologue of human GRB2-associated-binding protein 1 (GAB1) [12]. 

Solution NMR spectroscopy has been applied to investigate the biophysical properties of isolated Drk-NSH3 and Drk-CSH3. As mentioned earlier, the common 3D structure of SH3 domains consists of five β-strands. Apart from the other homologues, Drk-NSH3 shows exceptionality in structural behaviour which is in a dynamic equilibrium between the folded and unfolded states [13,14]. Drk-NSH3 binds to PRMs of Sos by recognising the motif of PxxPxR [11,15]. Reports of NMR titration experiments of Drk-NSH3 with a Sos-derived peptide, YRAVPPPLPPRR, showed that peptide interaction stabilises the folded state [14]. Unlike Drk-NSH3, our solution NMR study on Drk-CSH3 revealed that this domain exhibits only a folded state [16]. It has been proposed that Drk-CSH3 binds the PRMs with the PxxPxR motif in Sos and those with the PxxRxxKP motif in Dos [11,12]. Our previous NMR titration study with Drk-CSH3 with Sos- and Dos-derived peptides has revealed that Drk-CSH3 has a stronger affinity towards Sos [16]. 

Although the interaction between Drk-NSH3 and Sos-derived peptides has been investigated since then [17], the detailed interaction mechanism of Drk remains elusive due to limited information regarding, particularly, the interaction between Drk-NSH3 with the PRMs of Dos. To understand the multivalent interactions of Drk with Sos and Dos, a comprehensive analysis of the interactions between the isolated SH3 domains and the PRMs in Sos and Dos is necessary. We, therefore, conducted NMR titration experiments utilising peptides derived from Sos and Dos and evaluated their binding affinity to both SH3 domains. Furthermore, docking simulations were employed to explore the structural orientation of the peptides in relation to the SH3 domains. 

## 2. Results

### 2.1. The NMR Titration Experiments of Two SH3 Domains of Drk with Sos- and Dos-Derived Peptides

*Drosophila* Sos is composed of four folded domains (a Dbl-homology domain, a pleckstrin-homology (PH) domain, an N-terminal Ras-GEF domain and an Ras-GEF). In addition, Sos possesses an intrinsically disordered C-terminal region (residues 1067–1596), which contains two PRMs with the PxxPxR motif (Figure 1b) [11]. In contrast, Dos has one folded domain (a PH domain), which is followed by a C-terminal unstructured region (residues 114–773) containing two PRM with the PxxxRxxKP motif (Figure 1c) [12].

A series of multi-point NMR titrations were performed to determine the dissociation constants (*K*_d_s) of Sos- or Dos-derived peptides for the two SH3 domains and to identify potential binding sites on the protein surface. We used two peptides from Sos, YRAVPPPLPPRR, corresponding to residues 1339–1350 (henceforth referred to as Sos-S1), and GELSPPPIPPRL, corresponding to residues, 1377–1388 (henceforth referred to as Sos-S2), as well as two peptides from Dos, DCPPVNRKLKPKV, corresponding to residues 638–650 (henceforth referred to as Dos-S1), and GPPSVDRKCKPNA, corresponding to residues 690–702 (henceforth referred to as Dos-S2). The titration experiments were carried out by repeating the measurement of 2D ^1^H-^15^N HSQC spectra of either ^15^N-labelled Drk-NSH3 or ^15^N-labelled Drk-CSH3 with a stepwise increase in the peptide concentration. The backbone resonance assignments of isolated Drk-NSH3 and the overlays of 2D ^1^H-^15^N HSQC spectra for each titration experiment are shown in Supplementary Figures S1–S7. The titration experiments of Drk-CSH3 with Sos-S1 and Sos-S2 have already been performed in our previous study [16].

In the case of Drk-NSH3, as Zhang et al. have reported previously [13], two cross peaks corresponding to the folded and unfolded states were initially shown for each residue (Supplementary Figure S1). As the peptide concentration was increased, the following changes were observed: (i) a decrease in the intensity of the cross peaks corresponding to the unfolded state; (ii) concentration-dependent chemical shift changes for the cross peaks corresponding to the folded state. On the other hand, Drk-CSH3 displayed only one set of cross peaks, corresponding to the folded state, for each residue in the 2D ^1^H-^15^N HSQC spectra.

**Figure 2:**
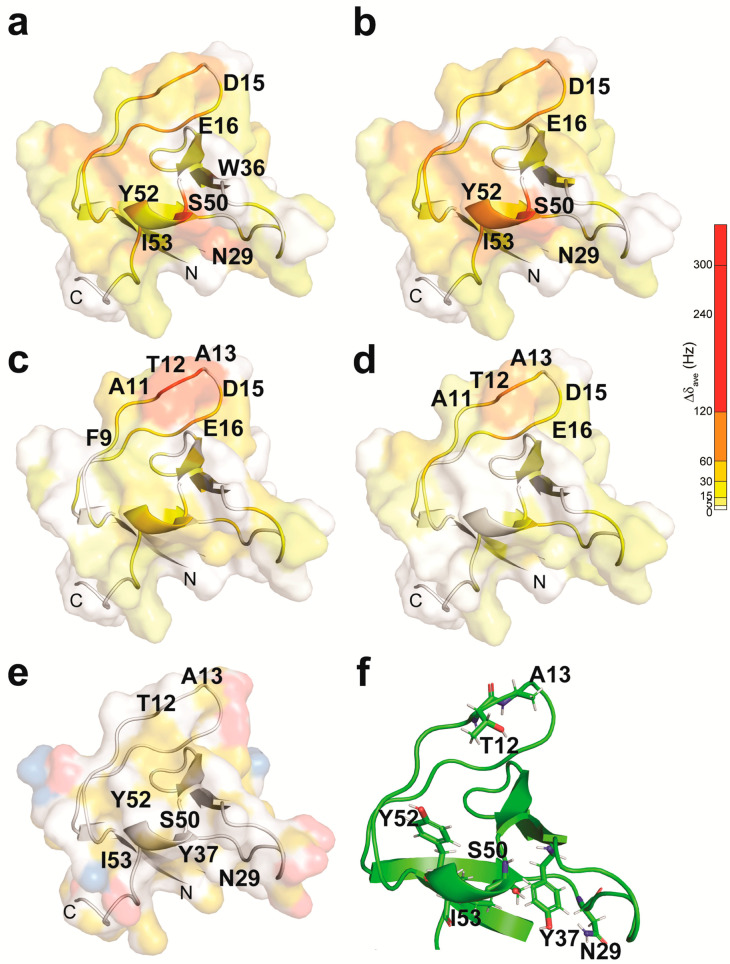
Chemical shift perturbation upon the titration with Sos-S1 (**a**), Sos-S2 (**b**), Dos-S1 (**c**) and Dos-S2 (**d**) represented on the solution structure of Drk-NSH3 (PDB ID: 2A36). The residues which were affected during the titration (with a protein:peptide mixing ratio of 1:8) are shown with a colour gradation from white (the lowest) to red (the highest). (**e**) The residues which showed significant chemical shift perturbations are indicated on the protein surface with the colour coding according to the hydrophobicity (yellow) and the electrostatic potential (negative: red; positive: blue). (**f**) The side chains of these residues are also shown.

**Figure 3:**
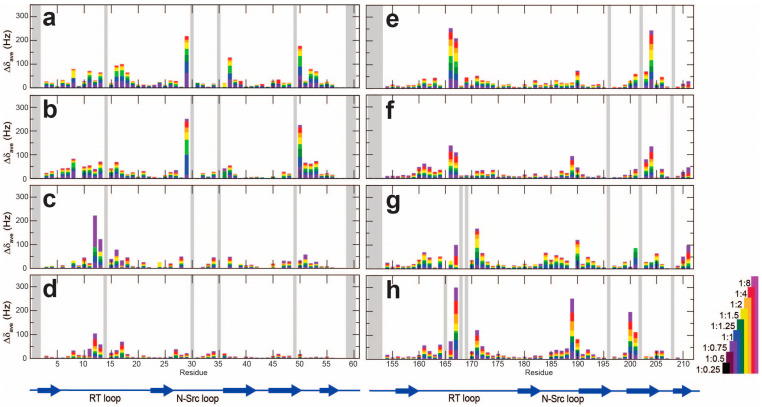
Plots of chemical shift perturbation of backbone ^1^H^N^ and ^15^N nuclei of Drk-NSH3 (**a**–**d**) and Drk-CSH3 (**e**–**h**) upon titration with Sos-S1 (**a**,**e**), Sos-S2 (**b**,**f**), Dos-S1 (**c**,**g**) and Dos-S2 (**d**,**h**). The mean shift difference Δδ_ave_ was calculated as [(Δδ^1^H^N^)^2^ + (Δδ^15^N)^2^]^1/2^, where Δδ^1^H^N^ and Δδ^15^N are the chemical shift differences (Hz) between Drk-NSH3 or Drk-CSH3 on its own and in the presence of peptides. The bar graphs are colour-coded according to the protein:peptide concentration ratio and are overlaid. The proline residues as well as the residues for which ^1^H^N^-^15^N correlation cross peaks were not analysed due to signal overlap or other reasons are shown in grey. The secondary structures of Drk-NSH3 and Drk-CSH3 are also shown.

Observed chemical shift perturbations (CSPs) were mapped onto the surface of the solutions structures of Drk-NSH3 (PDB ID: 2A36) [18] (Figure 2) or Drk-CSH3 (PDB ID: 7Y4N) [16] (Supplementary Figure S8). CSPs for each residue at each titration point were plotted as bar graphs (Figure 3).

The tendency of CSPs found for Drk-NSH3 can be summarised as follows. Based on other studies of SH3-PRM interactions, it was assumed that the residues F9, W36, L47, P49 and Y52 constitute the hydrophobic interaction surface of the Drk-NSH3. Excluding proline, the residues Y52 and L47 displayed moderate but clear CSPs for Sos-S1, S2 and Dos-S1, as we expected. These two residues likely contribute to the interactions with these peptides. We were not able to unambiguously distinguish CSPs or changes in peak intensity for the backbone of residues F9 and W36 due to severe signal overlaps. However, the H^ε1^ signals from the W36 side chain showed significant CSPs or reductions in peak intensity depending on the peptides (Supplementary Figures S2–S5), indicating that this residue indeed contributes to the interaction with all the peptides. Therefore, we can confirm that at least W36, L47 and Y52 contribute to the interactions with Sos or Dos. A long loop between the first and second β-strands (RT loop), particularly residues D15 and E16, showed large CSP in all four titration experiments. Interestingly, Sos-derived peptides showed significant CSPs for residues N29, S50, Y52 and I53, whereas Dos-derived peptides do not. In contrast, Dos-derived peptides showed an effect at residues A11, T12 and A13, whereas Sos-derived peptides do not. The location of these residues is mapped onto the structure, where the protein surface is colour-coded according to the hydrophobicity and electrostatics (Figure 2e). 

In our previous paper on Drk-CSH3, we used peptides of nine residues for each of Dos-S1 and Dos-S2 (PPVNRKLKP (640–648) and PSVDRKCKP (692–700), respectively). Both of the peptides showed rather weak affinity for Drk-CSH3 with the dissociation constants (*K*_d_s) of ~1.4 mM (Dos-S1) and ~4.8 mM (Dos-S2). In this study, peptides consisting of thirteen amino acid residues, extended by two residues each on the N- and C-termini, were used as “longer” Dos-S1 and Dos-S2 to examine the contribution of residues located outside the PxxxRxxKP motif. 

The *K*_d_ values for each peptide were calculated based on the CSP data. Zhang et al. have discussed the binding of Drk-NSH3 to the Sos-S1 peptide using a fold/unfold equilibrium model, where the unfolded state has no affinity for the peptide, and only the folded state can bind to it [19]. This is essentially identical to the commonly accepted conformational selection model, illustrating a scenario in which bound and unfolded conformations coexist in the free state, but only the bound state is chosen upon ligand contact. In Drk-NSH3, the fold and unfold conformations are in equilibrium in the free state, and the proportion of the folded conformation increases due to the exclusive binding of the Sos- or Dos-derived peptides to the folded form. Thus, similar to Zhan et al., we employed the conformational selection model, incorporating the equilibrium constant (*K*_f_) between the folded and unfolded states, as well as *K*_d_. To determine the *K*_d_ and *K*_f_ values of Drk-NSH3 concerning the Sos- and Dos-derived peptides, the spectra of Drk-NSH3 from a single titration series were fitted to the conformational selection model by simulating the CSPs along with the peptide additions using non-linear least-squares fitting.

For Drk-CSH3, a straightforward single-binding model was adopted, as there was no observation of structural equilibrium before or after the binding. In both models, to ensure the reliability of the parameter estimates, a bootstrapping method was employed with 1000 iterations. 

The calculated *K*_d_ values are summarised in Table 1. First, the combination of Drk-NSH3 with Sos-derived peptides showed the strongest affinity (Sos-S1: ~13 μM; Sos-S2: ~41 μM). The *K*_d_ difference between Sos-S1 and Sos-S2, despite having the same number of proline residues according to the PxxPxR motif, suggests that arginine residues (Sos-S1 has two extra arginine residues in addition to the one in the motif) partially contribute to the affinity. This is supported by the following results showing that relatively large CSPs were observed for the negatively charged residues in the RT-loop regions (residues D14, D15 and E16). A similar observation has been reported for the N-terminal SH3 domain of GRB2, which showed a higher affinity towards the PxxPxR motif. The peptide (PVPPPVPPRRRP) contains three arginine residues in the C-terminus, associating the negatively charged residues (D15 and E16) in the RT-loop region [6]. In contrast, Sos-S1 and Sos-S2 showed 10 times larger *K*_d_ values (Sos-S1: ~160 μM; Sos-S2: ~460 μM), to Drk-CSH3, suggesting that NSH3 is the initial binding site for Sos.

The “longer” Dos-S1 and Dos-S2 (Dos-S1: ~125 μM; Dos-S2: ~250 μM) showed stronger affinity to Drk-CSH3 than those of the “shorter” Dos peptides (Dos-S1: ~1500 μM; Dos-S2: ~5000 μM), indicating the additional residues outside the PxxxRxxKP motif contribute to the interaction. The *K*_d_ values of Dos-S1 and Dos-S1 with Drk-NSH3 were calculated to be ~80 μM and ~250 μM, respectively. It is very interesting that the Dos-derived peptides showed almost similar affinity for Drk-NSH3 and Drk-CSH3. This contrasts with the much stronger affinity of Sos-derived peptides to Drk-NSH3 compared to Drk-CSH3.

### 2.2. Docking Simulations of Sos and Dos-Derived Peptides on Drk-NSH3 and Drk-CSH3

The PRM-binding surface of the SH3 domain generally consists of a region composed of hydrophobic residues and surrounding negatively charged patches. The orientation of the PRM-peptide is, therefore, influenced by the presence of arginine and lysine residues contained in the sequence. To visualise the potential orientation of the peptides on the surface of the SH3 domains, docking simulations were performed using the AutoDock CrankPep 0.1 software [20]. The CSP data obtained from the NMR titration experiments were utilised to define the possible region of peptide-interacting surface on SH3 domains, which was created using Python molecular viewer (PMV) [21]. Proline-rich peptides orient on the SH3 domain basically in two manners, “Class I” and “Class II” [22]. The peptides with the PxxxRxxKP motif, however, have been reported to bind with neither a “Class I” nor “Class II” conformation, despite sharing the same binding interface [23].

**Figure 4:**
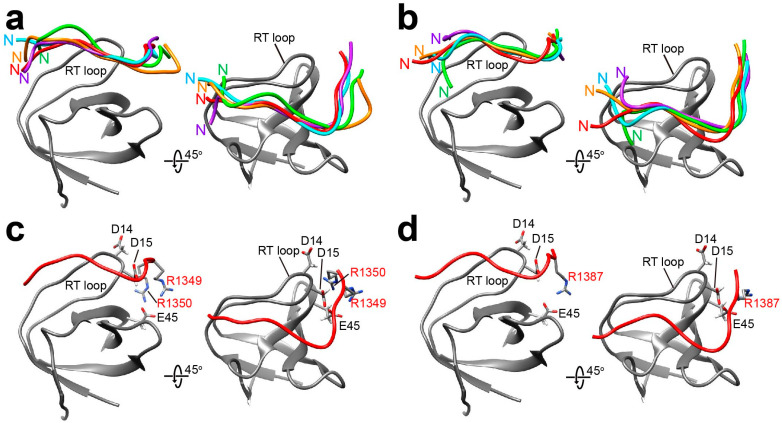
The models of the interactions of Drk-NSH3 with Sos-S1 (**a**,**c**) and Sos-S2 (**b**,**d**). In (**a**,**b**), the top five structures with the lowest energy are presented for each, in which the positions of the N-terminal of the peptides are indicated. In (**c**,**d**), relative positions of the arginine residues (annotated in red) in the peptides against the surrounding negatively charged residues of Drk-NSH3 are shown.

The docking simulation of Sos-derived peptides with the folded state of Drk-NSH3 showed the peptide orientation as a Class II type (Figure 4a,b). This is consistent with the tendency that peptides with typical PxxPxR motif will adopt in a Class II orientation manner on the SH3 domain surface. Our docking results of Sos-S1 (Figure 4a) showed that R1349 and R1350 showed more proximity towards the negatively charged cluster composed of D14 and D15 on the RT loop and E45 (Figure 4c). A similar result has been reported for the orientation of a peptide of VPPPVPPRRR against the N-terminal SH3 domain of GRB2 (PDB ID: 1AZE) [24]. The Sos-S2 peptide was similarly adopted for “Class II” orientation (Figure 4b), where the R1387 approaches the negatively charged cluster (Figure 4d). In our previous study on the interaction of Drk-CSH3 with PRMs, we found that the Sos-S1 exhibited a “Class I” orientation, while the Sos-S2 peptide adopted a “Class II” orientation [16]. The sequence comparison of Drk-NSH3 and Drk-CSH3 revealed that the hydrophobic residues are aligned similarly, whereas the charged residues are not (Supplementary Figure S9). As an example, in Drk-CSH3, the corresponding location for E45 in Drk-NSH3 is lysine. The difference in the binding mode of Sos-S1, which has the highest affinity to the SH3 domains, to Drk-NSH3 and Drk-CSH3 is probably due to electrostatic potential surface differences.

**Figure 5:**
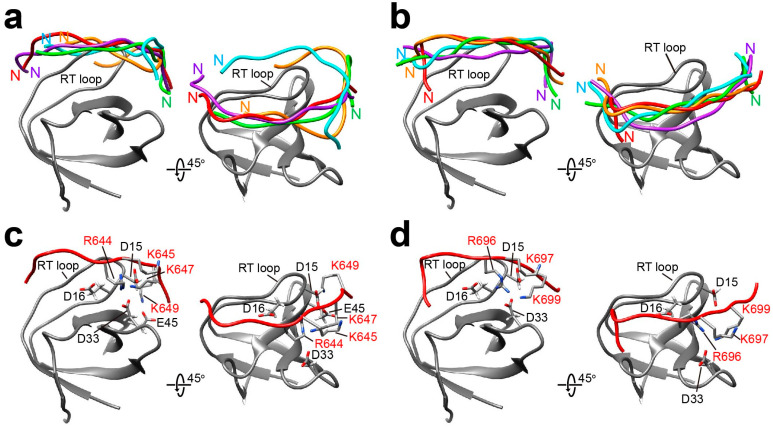
The models of the interactions of Drk-NSH3 with Dos-S1 (**a**,**c**) and Dos-S2 (**b**,**d**). In (**a**,**b**), the top five structures with the lowest energy are presented for each, in which the positions of the N-terminal of the peptides are indicated. In (**c**,**d**), relative positions of the lysine and arginine residues (annotated in red) in the peptides against the surrounding negatively charged residues of Drk-NSH3 are shown.

The docking simulations for Dos-derived peptides against Drk-NSH3 provided a mixed ensemble of both “Class I” and “Class II” polarities (Figure 5a,b). In both Dos-S1 and Dos-S2, the arginine in the PxxxRxxKP motif is immediately followed by a lysine residue. The representative structures (Figure 5c,d) show that the positively charged region in the peptides, which consists of the sequential arginine-lysine and the lysine residue in the motif, is in close proximity to the negatively charged region described above. 

In our previous report, the “short” Dos-derived peptides, consisting of nine residues, were not well positioned against Drk-CSH3 (Supplementary Figure S10c,d), presumably due to the weak affinity of the peptides for Drk-CSH3 [16]. However, docking simulations utilising the “longer” Dos-derived peptides showed relatively defined positions (Supplementary Figure S10a,b). Their orientations differ from both Class I and II and also from the positions reported in the crystal structures of the C-terminal SH3 domain of GRB2 with two human GRB2-associated binding protein 2 (GAB2)-derived peptides APPPRPPKP and IQPPPVNRNLKPDRK (PDB IDs: 2W0Z and 2VWF, respectively) [25]. On the other hand, there is a consistent trend for the positively charged side chains of lysine and arginine residues in the PxxxRxxKP motif to be located near the glutamate residues in the RT loop. Since the N- and C-terminal extensions also significantly affect the calculated *K*_d_s, the obtained positions of Dos-derived peptides on Drk-CSH3 will need to be verified in the future.

## 3. Discussion

Approximately 40% of the eukaryotic proteome is composed of intrinsically disordered proteins (IDPs) or IDRs, many of which play important roles in signal transduction [26,27]. In the case of *Drosophila*, the interactions between Drk with Sos and Dos are crucial for the signal transduction pathways; therefore, the functionality of the SH3 domains and their site-specific interactions play vital roles.

**Figure 6:**
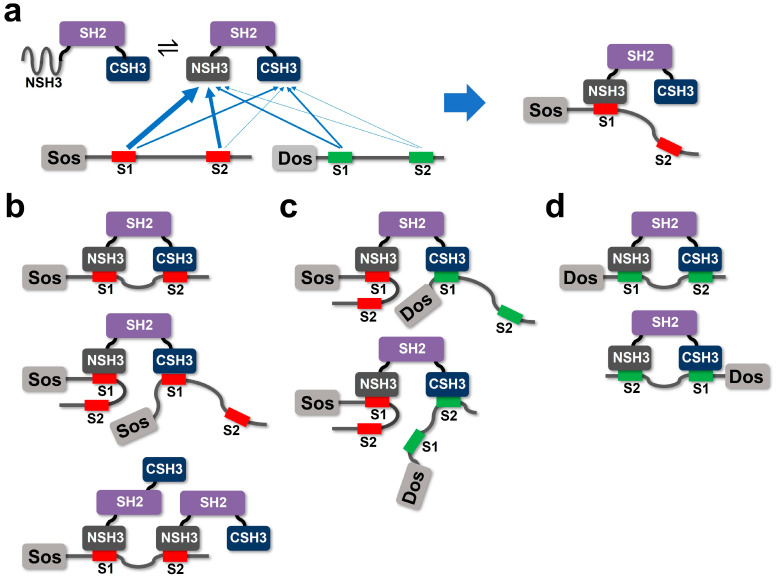
Schematic illustrations of the interactions of Drk with Sos and Dos. (**a**) Relative affinities between each SH3 domain and the PRMs of Sos and Dos are represented schematically by the thickness of the blue arrows (the combination of Sos-S1 and NSH3 has the highest affinity). (**b**) Potential interaction patterns when only Sos molecules are present around Drk. (**c**) Potential interaction patterns when both Sos and Dos are present around Drk. (**d**) Potential interaction patterns when only Dos molecules are present around Drk.

In our study, we employed NMR titration experiments to investigate the site-specific interactions of Drk-NSH3 and Drk-CSH3 with the PRMs derived from Sos and Dos. The CSP patterns observed in both SH3 domains suggest that the mode of interaction is different for Sos and Dos. In summary, the *K*_d_ values obtained in this study show that the combination of Sos-S1 and Drk-NSH3 has the highest affinity (*K*_d_ = ~13 μM), followed by Sos-S2 and Drk-NSH3 (*K*_d_ = ~41 μM). Then, the three combinations, Sos-S1 and Drk-CSH3, Dos-S1 and Drk-NSH3, and Dos-S1 and CSH3, have similar intermediate affinities (*K*_d_ = ~80–160 μM), while the rest of the combinations, Sos-S2 and CSH3, Dos-S1 and CSH3, and Dos-S2 and CSH3, have a weaker affinity (*K*_d_ = ~250–450 μM) (Figure 6a). It is interesting that binding occurs first in NSH3, even though the domain is expected to be in equilibrium between the folded and unfolded states. It is also very interesting that the two Sos-derived PRMs clearly favour NSH3 for binding, whereas the two Dos-derived PRMs show almost similar affinities for NSH3 and CSH3.

From the *K*_d_ values determined in this study, it can be imagined that when Drk interacts with Sos, NSH3 and Sos-S1 binding occurs first (Figure 6a). If only Drk and Sos interactions are considered, as happens after the binding of NSH3 and Sos-S1, then the binding of CSH3 and Sos-S2 can occur between the same molecules (Figure 6b). Of course, if there is an excess of Sos, another Sos might bind to the vacant CSH3 using Sos-S1. Alternatively, if there is an excess of Drk, the vacant Sos-S2 could attract the NSH3 of another Drk (Figure 6b). When both Sos and Dos are present, it is conceivable that Sos-S1 binds to NSH3, and then Dos binds to the vacant CSH3 via Dos-S1 (or Dos-S2) (Figure 6c). Thus, a single Drk molecule may be able to activate two signalling pathways simultaneously. Finally, if only Dos is present around Drk, there can be two possible binding modes due to the competitive affinity of Dos-S1 and Dos-S2 for NSH3 (Figure 6d). It is also possible that Drk binds Dos as a many-to-many binding mode, as considered for Drk-Sos interactions in Figure 6b. 

In the present study, we successfully investigated the interactions of Sos or Dos with the SH3 domains of Drk, particularly those of Dos-derived PRMs with NSH3. As mentioned in the introduction, it was previously thought that NSH3 contributed to the binding to Sos and CSH3 to Dos. However, our NMR titration experiments revealed that these interactions have a more intricate relationship with multiple recognition patterns. We believe that our data provide a more realistic explanation for the interactions. On the other hand, the residues outside the proline-rich motifs can have non-negligible effects on SH3-binding, as revealed in the analysis of the interaction between Dos-derived peptides and CSH3. Further analyses using longer peptide sequences or, if possible, the entire C-terminal unstructured regions of Sos and Dos are, therefore, awaited. The present analysis did not account for the influence of inter-domain interactions within the Drk molecule on its interactions with Sos and Dos. In addition to the experiments using isolated Drk-NSH3 and Drk-CSH3 as in the present study, an investigation using the full-length Drk is essential to achieve a comprehensive understanding of the Drk-regulated biological events in *Drosophila*.

## 4. Materials and Methods

### 4.1. Purification of Drk-NSH3 and Drk-CSH3

The gene encoding Drk-NSH3 (residues 1–59) was cloned into a pET-3a vector (Novagen, Madison, WI, USA), and the expression plasmid was introduced into *Escherichia coli* BL21 (DE3) star strain (Invitrogen, Waltham, MA, USA). 

Uniformly ^13^C and ^15^N-labelled Drk-NSH3 were prepared by growing the bacteria at 37 °C in an M9 medium containing 1 g/L ^15^NH_4_Cl and 2 g/L [^13^C_6_] D-glucose as the sole nitrogen and carbon sources, respectively. Uniformly ^15^N-labelled Drk-NSH3 was produced identically except using 4 g/L [^12^C_6_] D-glucose. At OD_600nm_ of ~0.5, protein expression was induced by the addition of isopropyl-β-D-thiogalactopyranoside. The purification procedures described below were carried out at 4 °C. The harvested cells were suspended in the lysis buffer (50 mM Tris-HCl (pH 7.5), 1 mM DTT and 1 mM EDTA), and disrupted by sonication for 30 min on ice. The cell debris was clarified by centrifugation at 60,000× *g* for 1 h. Drk-NSH3 was purified using a DEAE sepharose Fast Flow (Cytiva, Marlborough, MA, USA) anion exchange column pre-equilibrated with the lysis buffer. The protein was eluted using a 0–1 M NaCl gradient. The Drk-NSH3 containing fractions were collected and concentrated using an Amicon Ultra-15 centrifugal concentrator with a 3 kDa molecular size cut-off (Merck, Darmstadt, Germany). Further purifications of the samples were achieved using a Superdex 75 pg Hi-loadTM 16/600 (Cytiva, Marlborough, MA, USA) gel filtration column pre-equilibrated with the purification buffer (50 mM Tris-HCl (pH 7.5) and 1 mM DTT), and then using a Resource Q (Cytiva) anion exchange column pre-equilibrated with the purification buffer with a 0–1 M NaCl gradient.

The purification of ^15^N-labelled Drk-CSH3 was carried out by utilising the protocol reported previously [16]. 

The final fractions containing Drk-NSH3 or Drk-CSH3 were concentrated and dissolved in the NMR buffer (20 mM KH_2_PO_4_/K_2_HPO_4_ (pH 7.4), 100 mM KCl, 1 mM DTT and 0.05% NaN_3_) including 10% D_2_O for NMR lock.

### 4.2. NMR Spectroscopy

All NMR experiments were performed on a Bruker Avance-III HD 600 MHz spectrometer at 25 °C. All NMR spectra were processed using the Azara 2.8 software (Wayne Boucher and Department of Biochemistry, The University of Cambridge). All spectra were visualised and analysed using the CcpNmr Analysis 2.5.1 software [28]. 

The assignments of ^1^H^N^-^15^N correlation cross peaks for Drk-NSH3 in the 2D ^1^H-^15^N HSQC spectra were transferred from the BMRB database (Entry 51327), which was confirmed by analysing 3D triple-resonance spectra measured on the ^13^C/^15^N-labelled Drk-NSH3. A series of 2D ^1^H-^15^N HSQC spectra were measured for titration experiments of ^15^N-labelled Drk-NSH3 (0.3 mM) in the presence of peptides derived from Sos, YRAVPPPLPPRR (Sos-S1) and GELSPPPIPPRL (Sos-S2), or Dos, DCPPVNRKLKPKV (Dos-S1) and GPPSVDRKCKPNA (Dos-S2), which were purchased from Eurofins Genomics, Tokyo, Japan. The experiments were performed in the NMR buffer at 25 °C and the peptide concentration was increased stepwise (for Sos-S1 and Sos-S2, protein/peptide molar ratios of 1:0.25, 1:0.5, 1:0.75, 1:1, 1:1.25, 1:1.5, 1:2, 1:2.5 1:3 1:3.5, 1:4, 1:4.5 and 1:8 were used, while for Dos-S1 and Dos-S2, protein/peptide molar ratios of 1:0.25, 1:0.5, 1:0.75, 1:1, 1:1.25, 1:1.5, 1:2, 1:2.5, 1:3, 1:3.5, 1:4 and 1:8 were used).

The titration experiments of ^15^N-labelled Drk-CSH3 with the “longer” Dos-derived peptides were performed based on the resonance assignments obtained previously [16]. A series of 2D ^1^H-^15^N HSQC spectra were measured for ^15^N-labelled Drk-CSH3 (0.3 mM) in the presence of DCPPVNRKLKPKV (Dos-S1) or GPPSVDRKCKPNA (Dos-S2). The peptide concentration was increased stepwise (for Sos-S1 and Sos-S2, protein/peptide molar ratios of 1:0.25, 1:0.5, 1:0.75, 1:1, 1:1.25, 1:1.5, 1:2, 1:2.5 1:3 1:3.5, 1:4 and 1:8 were used, while for Dos-S1 and Dos-S2, protein/peptide molar ratios of 1:0.25, 1:0.5, 1:0.75, 1:1, 1:1.25, 1:1.5, 1:2,1:2.5,1:3,1:3.5, 1:4 and 1:8 were used).

For the single-state model (Drk-CSH3), the *K*_d_s against the Sos- or Dos-derived peptides were calculated by non-linear regression analysis with the equation [29]:

$${∆}_{obs}={∆}_{max}\frac{\left(K_{d}+\left[L\right]+\left[P\right]\right)-\sqrt{{\left(K_{d}+\left[L\right]+\left[P\right]\right)}^{2}-4\left[L\right]\left[P\right]}}{2\left[P\right]}$$

where ${∆}_{obs}$ is the observed chemical shift perturbation, ${∆}_{max}$ is the maximum chemical shift perturbation and [*L*] and [*P*] are the ligand and protein concentrations, respectively. For the conformational selection model (Drk-NSH3), the *K*_d_s and *K*_f_s between the folded and unfolded states were calculated by non-linear regression analysis with the equation.

$${∆}_{obs}={∆}_{max}\frac{\left(A+\left[L\right]+\left[P\right]\right)-\sqrt{{\left(A+\left[L\right]+\left[P\right]\right)}^{2}-4\left[L\right]\left[P\right]}}{2\left[P\right]}$$

$$A=\frac{K_{d}\left(1+K_{f}\right)}{K_{f}}$$

In both models, the non-linear least-squares fitting was performed by the Levenberg–Marquardt algorithm. The precision of the model parameters was assessed by conducting the bootstrap resampling on the residuals of the regression model. This procedure was repeated 1000 times to generate the bootstrap distribution of the model parameters. The bootstrap confidence intervals of the model parameters were computed with the percentile method, i.e., the 25th and the 975th smallest values of the bootstrap distribution were regarded as the lower and the upper limits of the 95% bootstrap confidence intervals, respectively.

### 4.3. Molecular Docking

To explore the binding mode of the Sos- and Dos-derived peptides on the surface of the SH3 domain, ab initio modelling was performed using the AutoDock CrankPep (ADCP) 0.1 software [20]. The first step involved defining a grid box that covered the region where the chemical shift perturbation was observed. This was accomplished using the Python molecular viewer PMV 1.5.7 [21]. 

For Drk-NSH3 (PDB ID-2A36), the grid dimensions were set to 72 × 44 × 58 Å^3^, with a spacing of 0.375 Å. The centre coordinates for the grid were specified as 5.361, −4.222 and 4.611 in the *x*, *y* and *z* directions, respectively. Similarly, for Drk-CSH3 (PDB ID-7Y4N), the grid dimensions were set to 74 × 56 × 42 Å^3^, with the same spacing as Drk-NSH3. The centre coordinates for the grid were specified as 0.972, 1.444 and 3.222.

The calculations were performed with 2000 runs in 200,000 steps, allowing for a comprehensive exploration of the peptide binding modes on the SH3 domain surfaces.

## Figures and Tables

**Table 1:** The calculated dissociation constants (*K*_d_s) for Sos- and Dos-derived peptides. For the binding with Drk-NSH3, the calculated equilibrium constants (*K*_f_) between the folded and unfolded states are also shown.

Peptide	Sequence	Motif	Drk-NSH3	Drk-CSH3
Sos-S1	YRAVPPPLPPRR (1339–1350)	PxxPxR	13.24 ± 0.704 μM (*K*_f_ = 0.9589 ± 0.0044)	157.57 ± 8.405 μM
Sos-S2	GELSPPPIPPRL (1377–1389)	PxxPxR	41.44 ± 8.429 μM (*K*_f_ = 0.8813 ± 0.0435)	457.40 ± 17.334 μM
Dos-S1	DCPPVNRKLKPKV (638–650)	PxxxRxxKP	82.89 ± 4.535 μM (*K*_f_ = 0.4741 ± 0.026)	124.73 ± 14.724 μM
Dos-S2	GPPSVDRKCKPNA (690–702)	PxxxRxxKP	242.11 ± 11.608 μM (*K*_f_ = 0.6154 ± 0.0178)	250.17 ± 21.498 μM

## Supplementary Materials

The supporting information can be downloaded at: https://www.mdpi.com/article/10.3390/ijms241814135/s1.
