# Supplementary material for: Interactions of the N- and C-Terminal SH3 Domains of Drosophila Drk with the Proline-Rich Peptides from Sos and Dos
Source: Int J Mol Sci. 2023 Sep 15;24(18):14135. doi: 10.3390/ijms241814135 (PMC10532153; doi:10.3390/ijms241814135)
Supplement: Supplementary file 1 [file ijms-24-14135-s001.zip › ijms-2597487-supplementary.pdf]

Supplementary data for:

Interactions of the N- and C-Terminal SH3 Domains of *Drosophila* Drk with the Proline-Rich Peptides from Sos and Dos

Pooppadi Maxin Sayeesh †, Mayumi Iguchi †, Yusuke Suemoto, Jin Inoue ‡, Kohsuke Inomata, Teppei Ikeya * and Yutaka Ito *

Department of Chemistry, Tokyo Metropolitan University, 1-1 Minami-Osawa, Hachioji, Tokyo 192-0373, Japan
*Authors to whom correspondence should be addressed.
†These authors contributed equally to this work.
‡Present address: Current address: Tohoku Medical Megabank Organization, and The Advanced Research Center for Innovations in Next-Generation Medicine (INGEM), Tohoku University, Sendai 980-8577, Japan

Table of Contents

Supplementary Figure S1. 2D ^{1}H-^{15}N HSQC spectra of ^{15}N-labelled Drk-NSH3.
Supplementary Figure S2. Overlays of 2D ^{1}H-^{15}N HSQC spectra from multipoint titrations of ^{15}N-labelled Drk-NSH3 with Sos-S1 (YRAVPPPLPPRR) peptide.
Supplementary Figure S3. Overlays of 2D ^{1}H-^{15}N HSQC spectra from multipoint titrations of ^{15}N-labelled Drk-NSH3 with Sos-S2 (GELSPPPIPPRL) peptide.
Supplementary Figure S4. Overlays of 2D ^{1}H-^{15}N HSQC spectra from multipoint titrations of ^{15}N-labelled Drk-NSH3 with Dos-S1 (DCPPVNRKLKPKV) peptide.
Supplementary Figure S5. Overlays of 2D ^{1}H-^{15}N HSQC spectra from multipoint titrations of ^{15}N-labelled Drk-NSH3 with Dos-S2 (GPPSVDRKCKPNA) peptide.
Supplementary Figure S6. Overlays of 2D ^{1}H-^{15}N HSQC spectra from multipoint titrations of ^{15}N-labelled Drk-CSH3 with Dos-S1 (DCPPVNRKLKPKV) peptide.
Supplementary Figure S7. Overlays of 2D ^{1}H-^{15}N HSQC spectra from multipoint titrations of ^{15}N-labelled Drk-CSH3 with Dos-S2 (GPPSVDRKCKPNA) peptide.
Supplementary Figure S8. Chemical shift perturbation of Sos- and Dos- derived peptides on Drk-CSH3.
Supplementary Figure S9. Sequence comparison of Drk-NSH3 and Drk-CSH3.
Supplementary Figure S10. The models of the interactions of Drk-CSH3 with the Dos-S1 (DCPPVNRKLKPKV) and Dos-S2 (GPPSVDRKCKPNA) peptides.

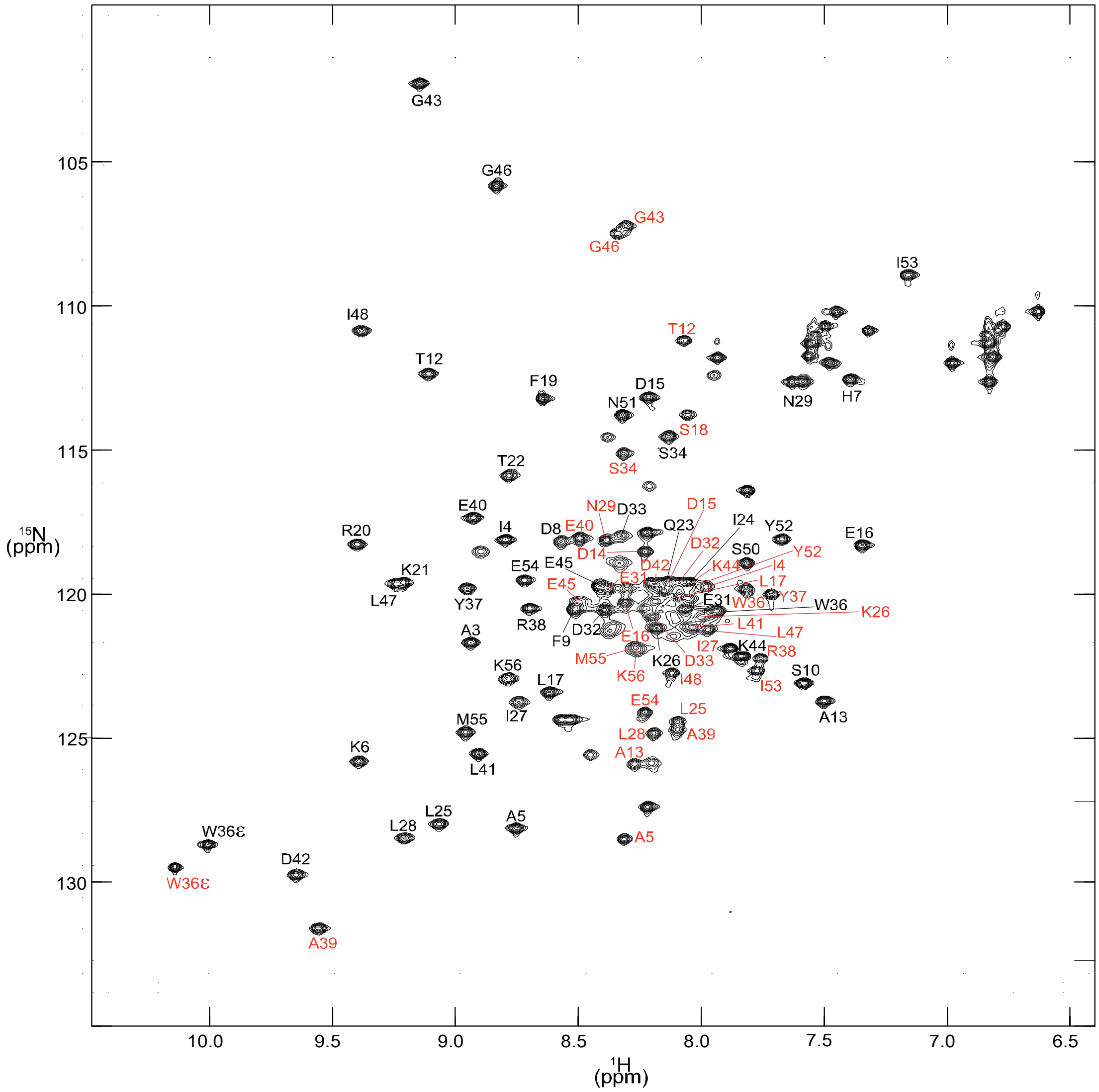

Supplementary Figure S1. The 2D ^{1}H-^{15}N-HSQC spectrum of Drk-NSH3. Fold and unfold (black and red) cross-peaks are labelled with their corresponding backbone assignments.

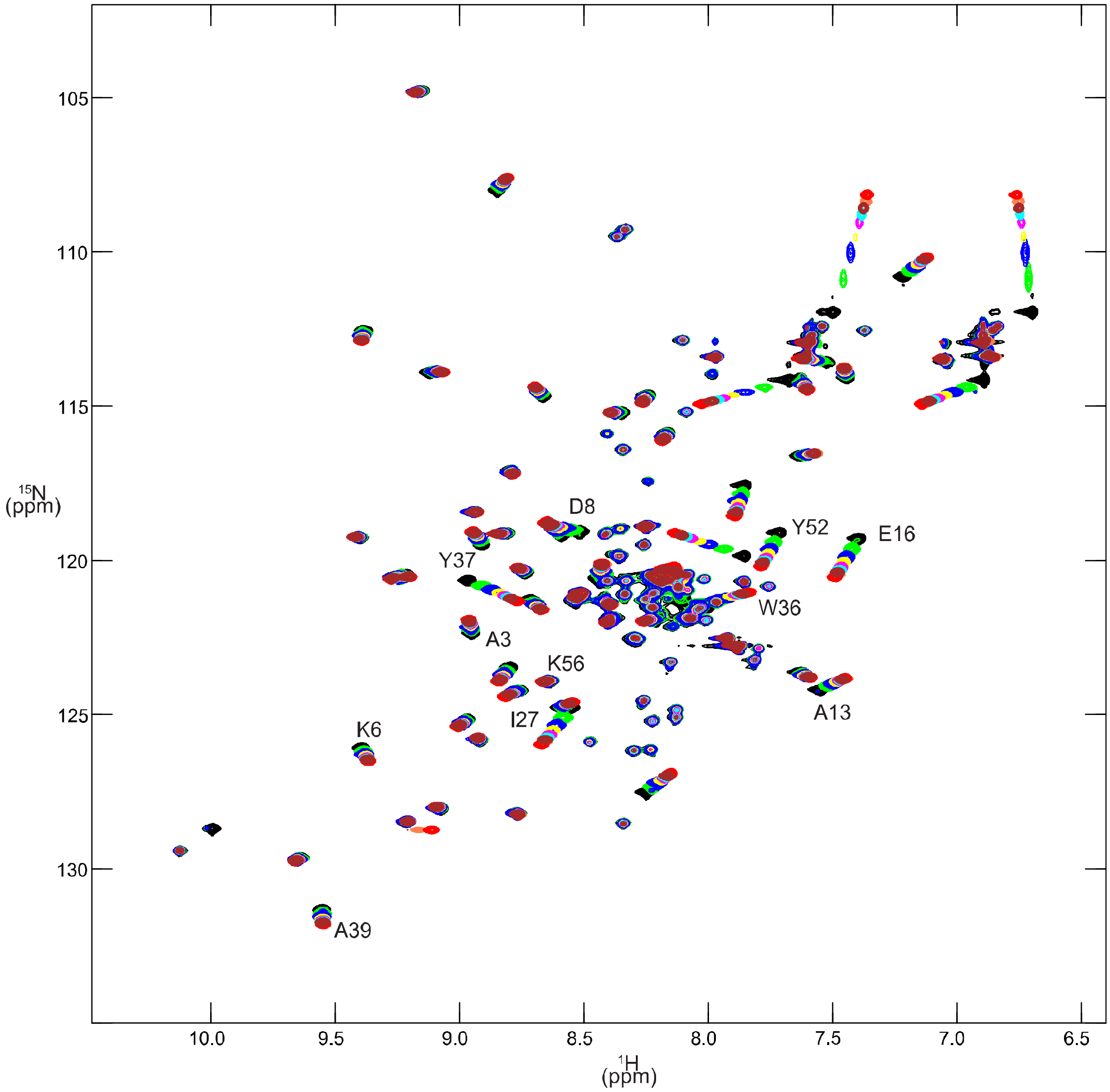

Supplementary Figure S2. Overlays of 2D ^{1}H-^{15}N HSQC spectra from multipoint titrations of ^{15}N-labelled Drk-NSH3 with Sos-S1 (YRAVPPPLPPRR) peptide. The peptide concentration was increased stepwise (the protein: peptide molar ratio of 1:0.25, 1:0.5, 1:075, 1:1, 1:1.25, 1:1.5, 1:2, 1:2.5, 1:3, 1:3.5, 1:4, 1:4.5 and 1:8). In this figure, the colour codes of ^{1}H-^{15}N correlation cross-peaks at each titration point, showing the molar ratio of Drk-NSH3: Sos-S1, are as follows: black (1:0); green (1:0.25); blue (1:0.5); yellow (1:0.75); magenta (1:1); cyan (1:1.25); brown (1:1.5); coral (1:2); red (1:4). Cross peaks that showed large chemical shift changes were annotated.

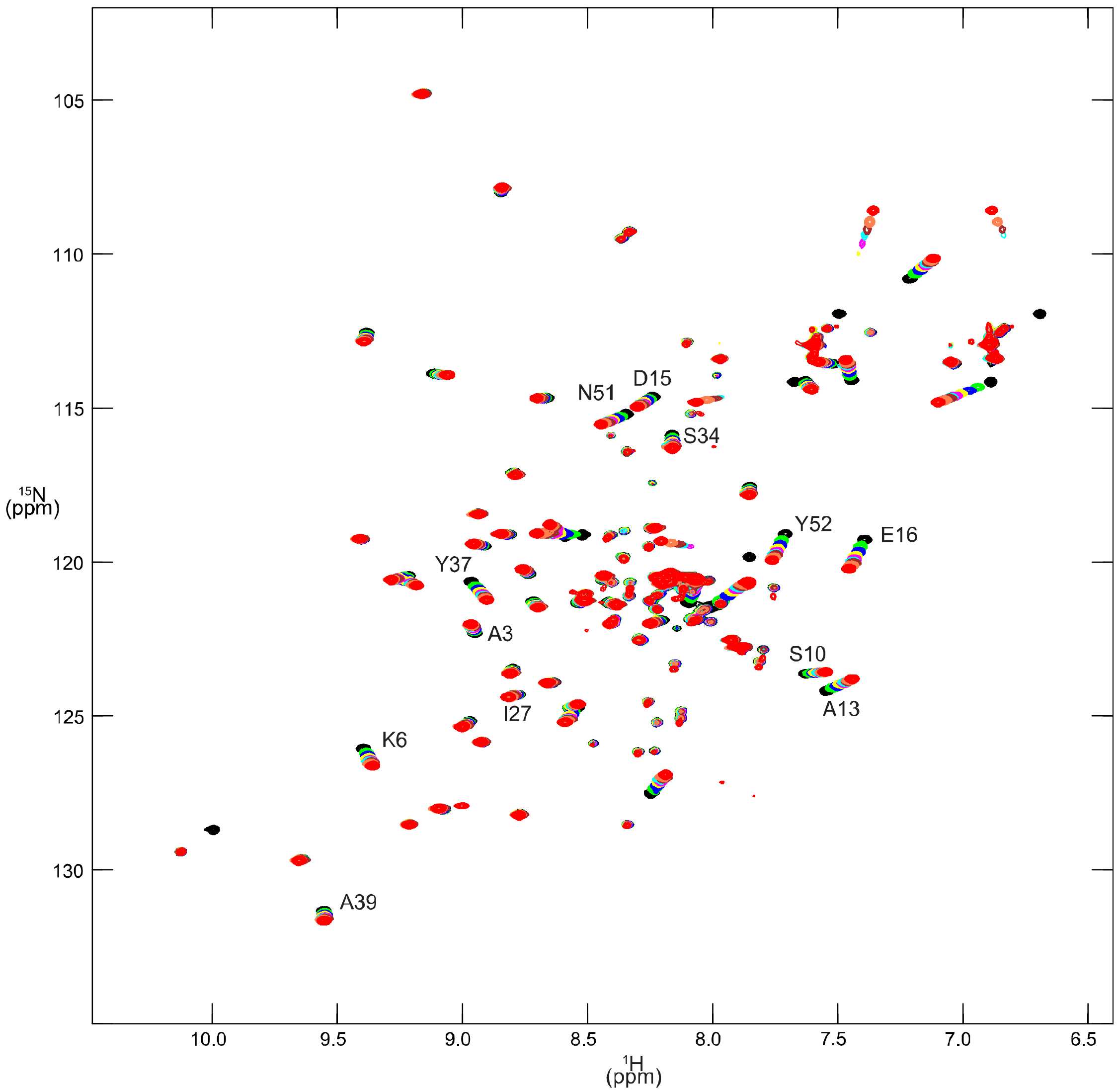

Supplementary Figure S3. Overlays of 2D ^{1}H-^{15}N HSQC spectra from multipoint titrations of ^{15}N-labelled Drk-NSH3 with Sos-S2 (GELSPPPIPPRL) peptide. The peptide concentration was increased stepwise (the protein:peptide molar ratio of 1:0.25, 1:0.5, 1:075, 1:1, 1:1.25, 1:1.5, 1:2, 1:2.5, 1:3, 1:3.5, 1:4, 1:4.5 and 1:8). In this figure, the colour codes of ^{1}H-^{15}N correlation cross-peaks at each titration point, showing the molar ratio of Drk-NSH3: Sos-S2, are as follows: black (1:0); green (1:0.25); blue (1:0.5); yellow (1:0.75); magenta (1:1); cyan (1:1.25); brown (1:1.5); coral (1:2); red (1:4). Cross peaks that showed large chemical shift changes were annotated.

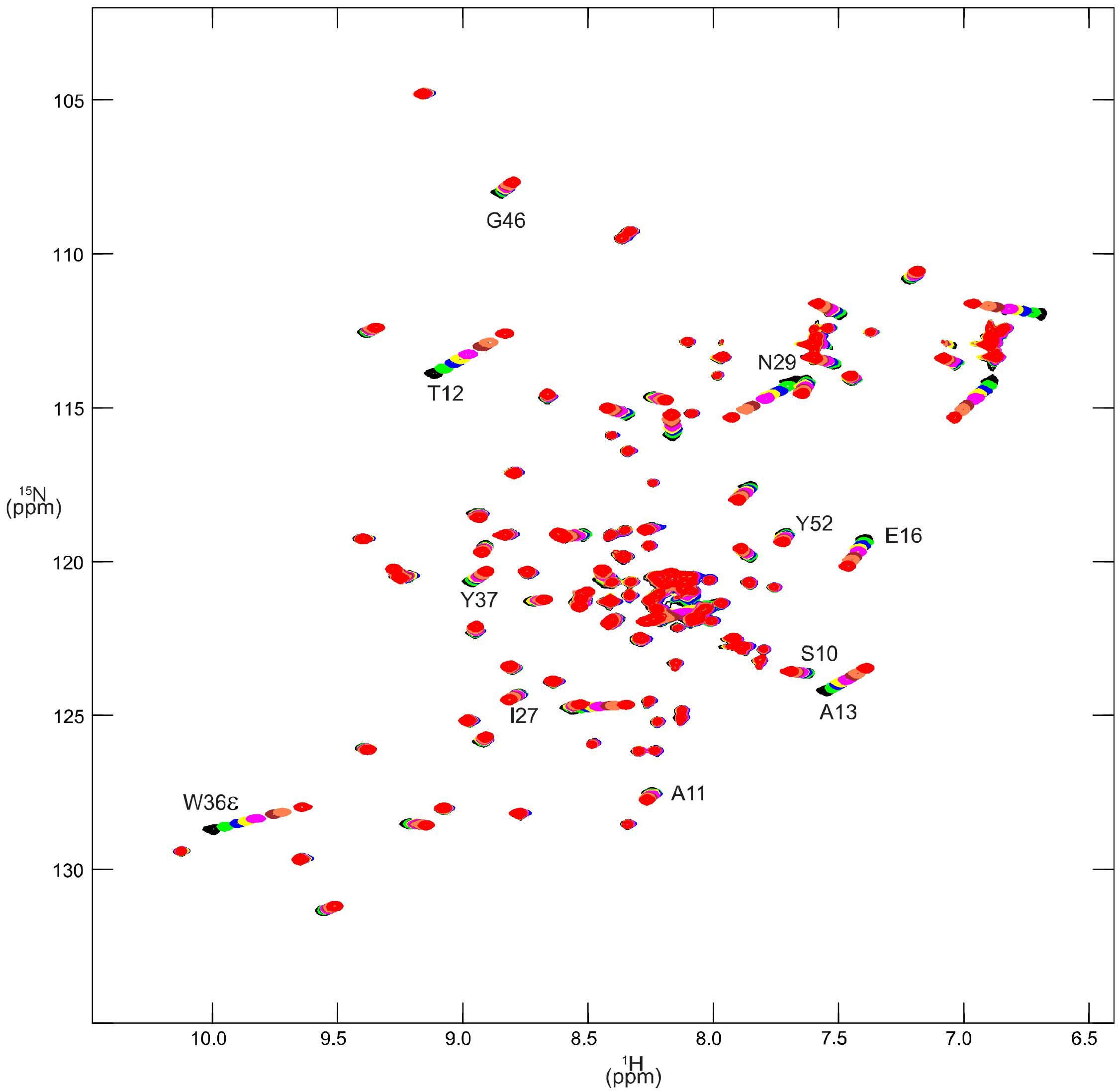

Supplementary Figure S4. Overlays of 2D ^{1}H-^{15}N HSQC spectra from multipoint titrations of ^{15}N-labelled Drk-NSH3 with Dos-S1 (DCPPVNRKLKPKV) peptide. The peptide concentration was increased stepwise (the protein:peptide molar ratio of 1:0.25, 1:0.5, 1:075, 1:1, 1:1.25, 1:1.5, 1:2, 1:2.5, 1:3, 1:3.5, 1:4 and 1:8). In this figure, the colour codes of ^{1}H-^{15}N correlation cross-peaks at each titration point, showing the molar ratio of Drk-NSH3: Dos-S1, are as follows: black (1:0); green (1:0.25); blue (1:0.5); yellow (1:0.75); magenta (1:1); cyan (1:1.25); brown (1:1.5); coral (1:2); red (1:4). Cross peaks that showed large chemical shift changes were annotated.

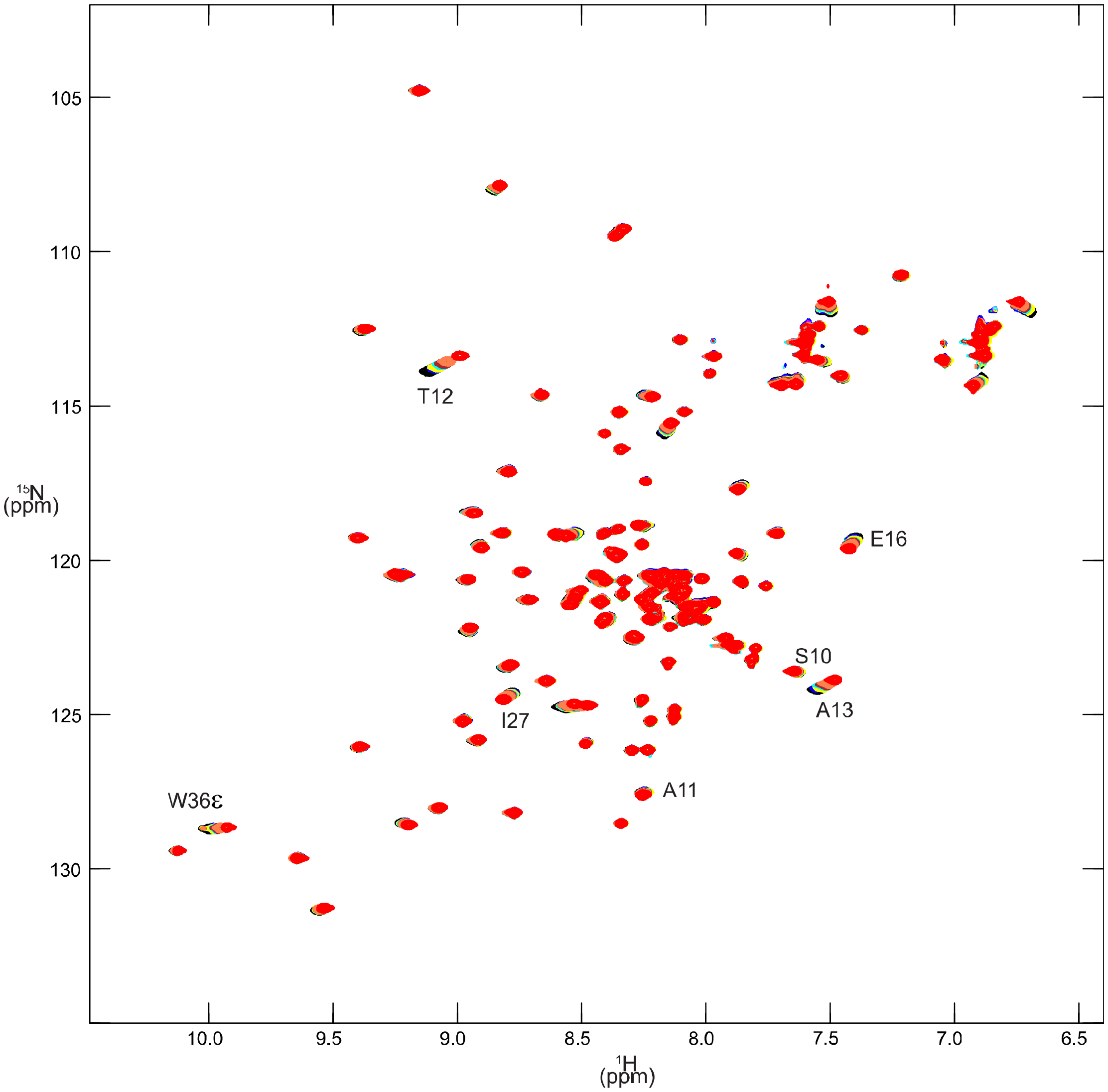

Supplementary Figure S5. Overlays of 2D ^{1}H-^{15}N HSQC spectra from multipoint titrations of ^{15}N-labelled Drk-NSH3 with Dos-S2 (GPPSVDRKCKPNA) peptide. The peptide concentration was increased stepwise (the protein:peptide molar ratio of 1:0.25, 1:0.5, 1:075, 1:1, 1:1.25, 1:1.5, 1:2, 1:2.5, 1:3, 1:3.5, 1:4 and 1:8). In this figure, the colour codes of ^{1}H-^{15}N correlation cross-peaks at each titration point, showing the molar ratio of Drk-NSH3: Dos-S2, are as follows: black (1:0); green (1:0.25); blue (1:0.5); yellow (1:0.75); magenta (1:1); cyan (1:1.25); brown (1:1.5); coral (1:2); red (1:4). Cross peaks that showed large chemical shift changes were annotated.

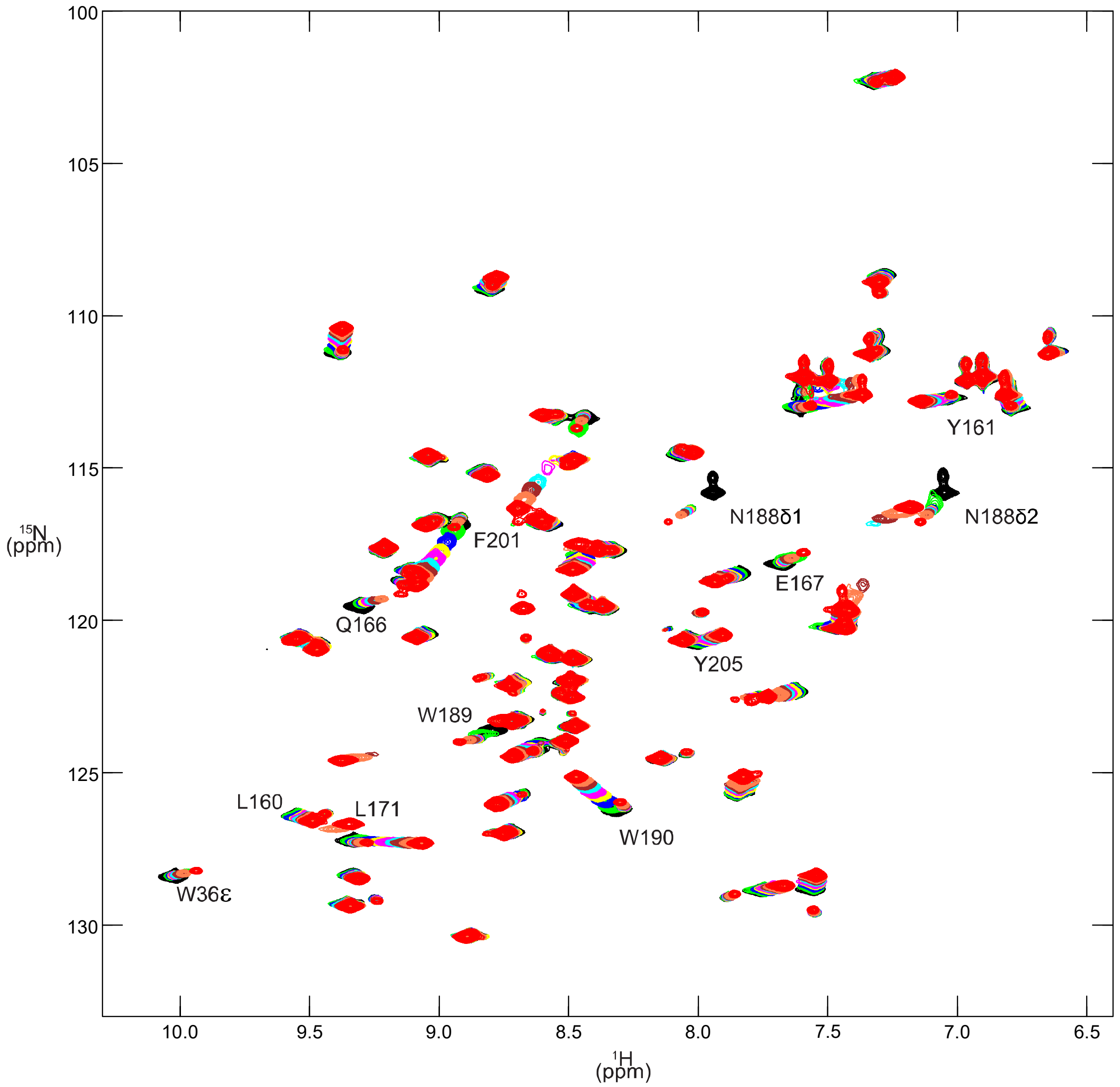

Supplementary Figure S6. Overlays of 2D ^{1}H-^{15}N HSQC spectra from multipoint titrations of ^{15}N-labelled Drk-CNSH3 with Dos-S1 (DCPPVNRKLKPKV) peptide. The peptide concentration was increased stepwise (the protein:peptide molar ratio of 1:0.25, 1:0.5, 1:075, 1:1, 1:1.25, 1:1.5, 1:2, 1:2.5, 1:3, 1:3.5, 1:4 and 1:8). In this figure, the colour codes of ^{1}H-^{15}N correlation cross-peaks at each titration point, showing the molar ratio of Drk-CSH3: Dos-S1, are as follows: black (1:0); green (1:0.25); blue (1:0.5); yellow (1:0.75); magenta (1:1); cyan (1:1.25); brown (1:1.5); coral (1:2); red (1:4). Cross peaks that showed large chemical shift changes were annotated.

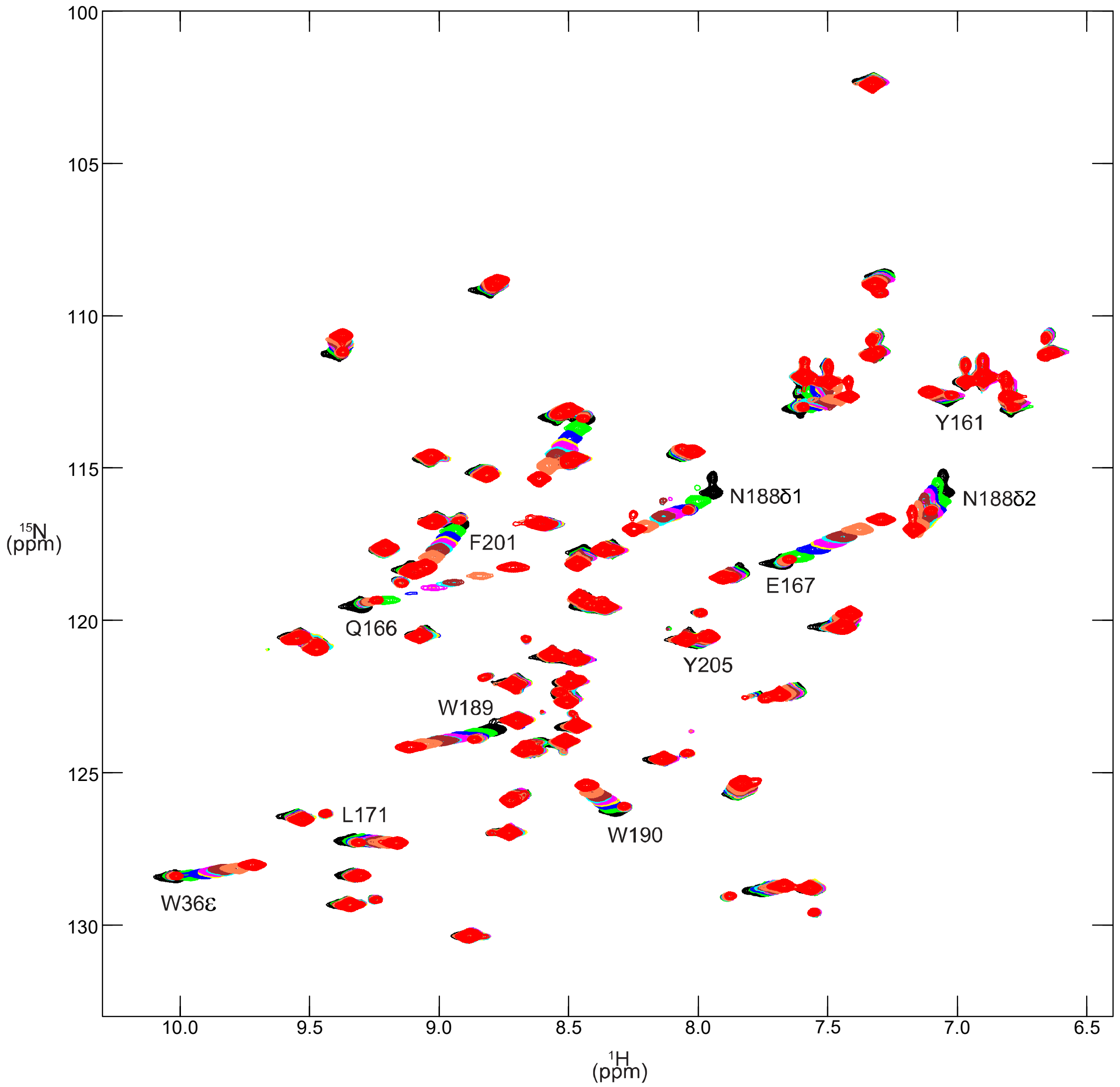

Supplementary Figure S7. Overlays of 2D ^{1}H-^{15}N HSQC spectra from multipoint titrations of ^{15}N-labelled Drk-CSH3 with Dos-S2 (GPPSVDRKCKPNA) peptide. The peptide concentration was increased stepwise (the protein:peptide molar ratio of 1:0.25, 1:0.5, 1:075, 1:1, 1:1.25, 1:1.5, 1:2, 1:2.5, 1:3, 1:3.5, 1:4 and 1:8). In this figure, the colour codes of ^{1}H-^{15}N correlation cross-peaks at each titration point, showing the molar ratio of Drk-CSH3: Dos-S2, are as follows: black (1:0); green (1:0.25); blue (1:0.5); yellow (1:0.75); magenta (1:1); cyan (1:1.25); brown (1:1.5); coral (1:2); red (1:4). Cross peaks that showed large chemical shift changes were annotated.

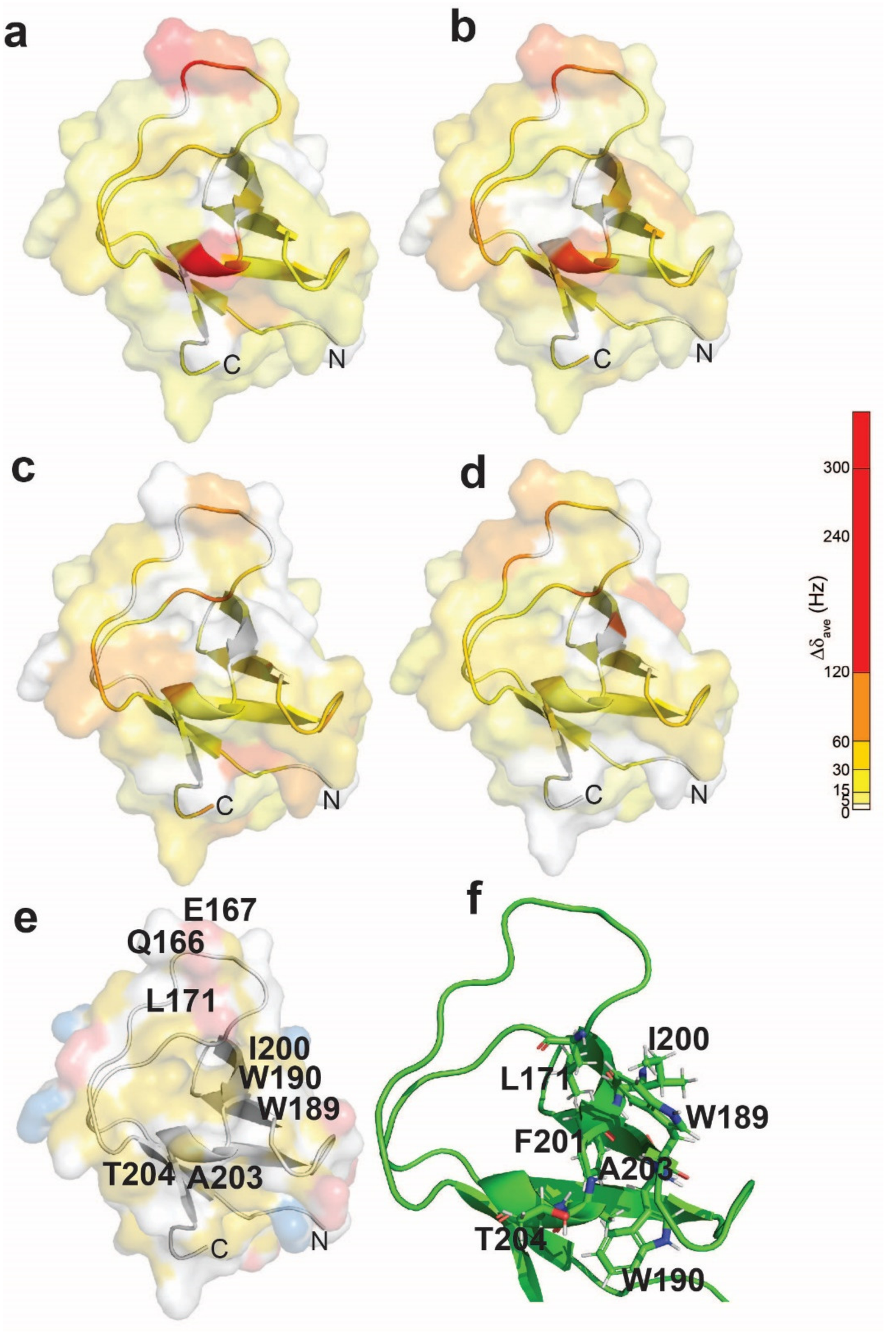

Supplementary Figure S8. Chemical shift perturbation upon the titration with Sos-S1 (**a**), Sos-S2 (**b**), Dos-S1 (**c**), and Dos-S2 (**d**) represented on the solution structure of Drk-CSH3 (PDB ID: 7Y4N). Figures **S9a** and **S9b** were created from the data obtained in our previous study[1]. The residues which were affected during the titration (with the protein:peptide mixing ratio of 1:8) are shown with a colour gradation from white (the lowest) to red (the highest). (**e**) The residues which showed significant chemical shift perturbations are indicated on the protein surface with the colour-coding according to the hydrophobicity (yellow) and the electrostatic potential (negative: red and positive: blue). (**f**) The sidechains of these residues are also shown.

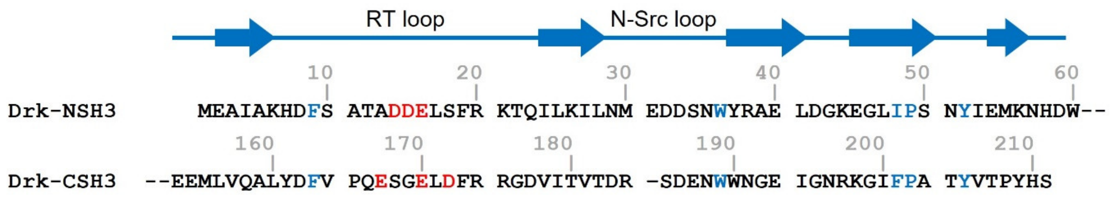

Supplementary Figure S9. Sequence comparison of Drk-NSH3 with the Drk-CSH3. The residues involved in the hydrophobic PRM-binding interface (blue) and the negatively charged residues in the RT loop (red) are indicated.

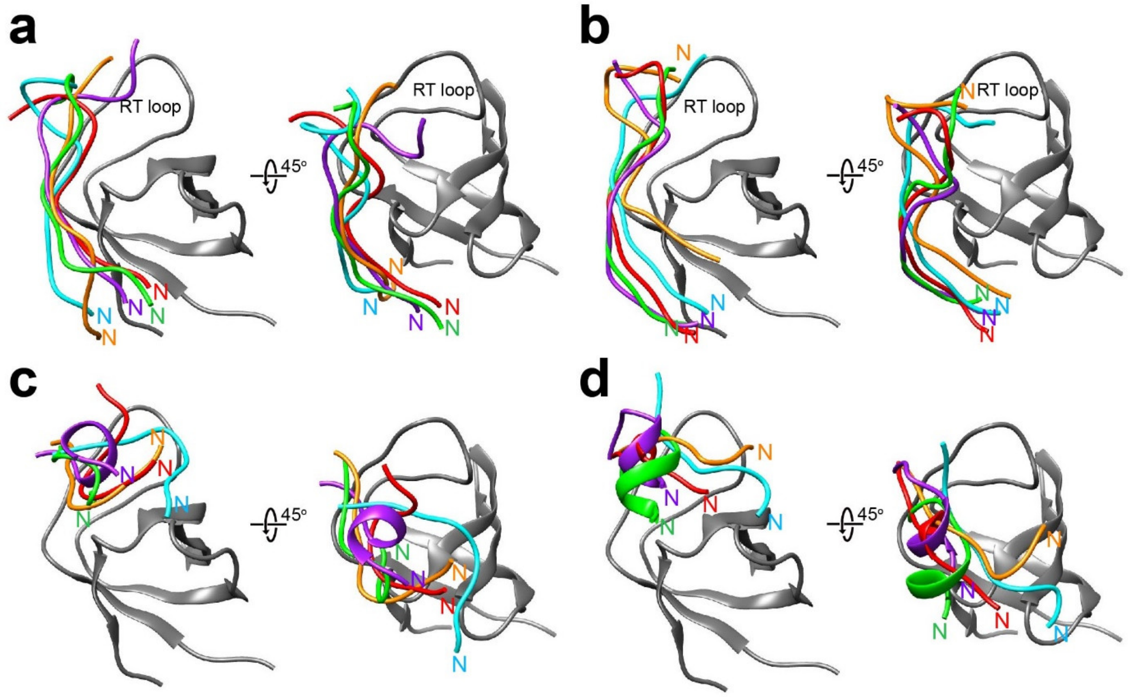

Supplementary Figure S10. The models of the interactions of Drk-CSH3 with the "longer" Dos-S1 (DCPPVNRKLKPKV) (**a**) and Dos-S2 (GPPSVDRKCKPNA) (**b**), which are compared with the results of docking simulations with the "shorter" Dos-S1 (PPVNRKLKP) (**c**) and Dos-S2 (PSVDRKCKP) (**b**) performed previously[1]. The top five structures with the lowest energy are presented for each, in which the positions of the N-terminal of the peptides are indicated.

1. Sayeesh, P. M.; Ikeya, T.; Sugasawa, H.; Watanabe, R.; Mishima, M.; Inomata, K.; Ito, Y., Insight into the C-terminal SH3 domain mediated binding of Drosophila Drk to Sos and Dos. *Biochemical and Biophysical Research Communications* **2022,** 625, 87-93.
